# Insights into canine reproductive health: ultrasonographic evaluation of the uterus—a review

**DOI:** 10.3389/fvets.2026.1717774

**Published:** 2026-01-20

**Authors:** Claudia Bracco, Alberto Contri, Sandra Goericke-Pesch

**Affiliations:** 1 Department of Veterinary Medicine, University of Teramo, Teramo, Italy; 2 Unit for Reproductive Medicine—Clinic for Small Animals, University of Veterinary Medicine Hannover, Hannover, Germany

**Keywords:** 3D and 4D ultrasonography, CEUS, dog, doppler, elastography, female reproductive system, ultrasonography, uterus

## Abstract

This review provides a comprehensive exploration of the applications and advancements in ultrasonographic techniques for evaluating the uterus in domestic canines. Conventional grey-scale ultrasonography remains a cornerstone for detecting anatomical and pathological changes, while emerging modalities such as Doppler ultrasonography, contrast-enhanced ultrasonography (CEUS), and ultrasound elastography (UEl) have expanded diagnostic capabilities by providing insights into vascularity, tissue stiffness, and microvascular perfusion. Recent innovations in three-dimensional (3D) and four-dimensional (4D) ultrasonography have further revolutionized imaging by enabling detailed visualization of fetal anatomy and dynamic intrauterine processes. While these techniques have been extensively studied in human and veterinary medicine for various organs, their application to the canine uterus, both non-pregnant and pregnant, remains underexplored. This review bridges gaps in existing literature by incorporating findings from related fields and outlining future directions for research to improve the diagnosis and management of reproductive uterine disorders in dogs.

## 1 Introduction

Ultrasonography is an essential diagnostic tool in veterinary medicine, offering real-time, non-invasive visualization of reproductive structures in domestic canines. Its role spans a wide array of applications, from monitoring normal physiological events such as pregnancy progression or fetal development evaluation to diagnosing and managing reproductive pathologies (1, 2). While grey-scale ultrasonography has been a reliable modality for decades, the advent of advanced imaging technologies has opened new possibilities. Doppler ultrasonography provides detailed evaluations of blood flow, offering critical insights into vascular health during different reproductive stages (2). Similarly, contrast-enhanced ultrasonography (CEUS), and ultrasound elastography (UEl) enable the assessment of microvascular perfusion and tissue elasticity, respectively, with significant implications for identifying pathological changes (3, 4). Lastly, 3D and 4D ultrasonography have been introduced, providing unique spatial and dynamic imaging of the uterus and fetuses (1).

This review summarizes both conventional and innovative ultrasound-based techniques applied to the evaluation of the canine uterus, providing an overview of the principles underlying each modality, the procedures used to examine the uterine structures, and the typical ultrasonographic features observed in physiological and pathological conditions. By integrating the available evidence, the review highlights the strengths and limitations of each technique and assists the clinician in selecting the most appropriate modality, or combination of modalities, to accurately characterize uterine disorders and support reproductive management in the bitch.

To compile the information presented, the scientific literature was surveyed using the main research databases, including PubMed, Scopus, Web of Science, and Google Scholar. Searches were conducted with broad species- and organ-related keywords (dog, bitch, uterus, reproductive tract, ultrasonography) and, depending on the imaging modality, specific terms (B-mode, Doppler, contrast-enhanced ultrasonography, CEUS, elastography, 3D ultrasound, 4D ultrasound). The time period considered ranged from 1980 to January 2025, with particular attention to studies published over the last fifteen years, reflecting the rapid evolution of diagnostic ultrasound technologies. All retrieved articles were examined, and the most relevant findings were included in the present review. When canine-specific studies were unavailable or scarce for a given technology, pertinent data from other domestic species or human medicine were consulted to provide comparative insights.

## 2 Uterine components evaluated by ultrasonography

### 2.1 Uterine tubes

The uterine tube (oviduct or fallopian tube) transports the ova from the ovary to the uterus. It is divided into four distinct sections: the infundibulum, which surrounds the ovary and is equipped with fimbriae to capture the ovum; the ampulla, the widest and most convoluted region where fertilization typically occurs; the isthmus, a narrower and more muscular segment that propels the fertilized ovum towards the uterus; and the uterotubal junction, which serves as the interface between the uterine tube and the uterus (5). The uterine tube’s wall consists of three main layers: the tunica mucosa, tunica muscularis, and tunica serosa (6). The mucosa contains ciliated and secretory epithelial cells that support gamete transport and early embryo viability, while the muscularis generates peristaltic movements. Functionally, the ampulla represents the main site of fertilization and provides a suitable microenvironment for sperm storage and capacitation and early embryo development (6, 7).

### 2.2 Uterus

**Figure 1:**
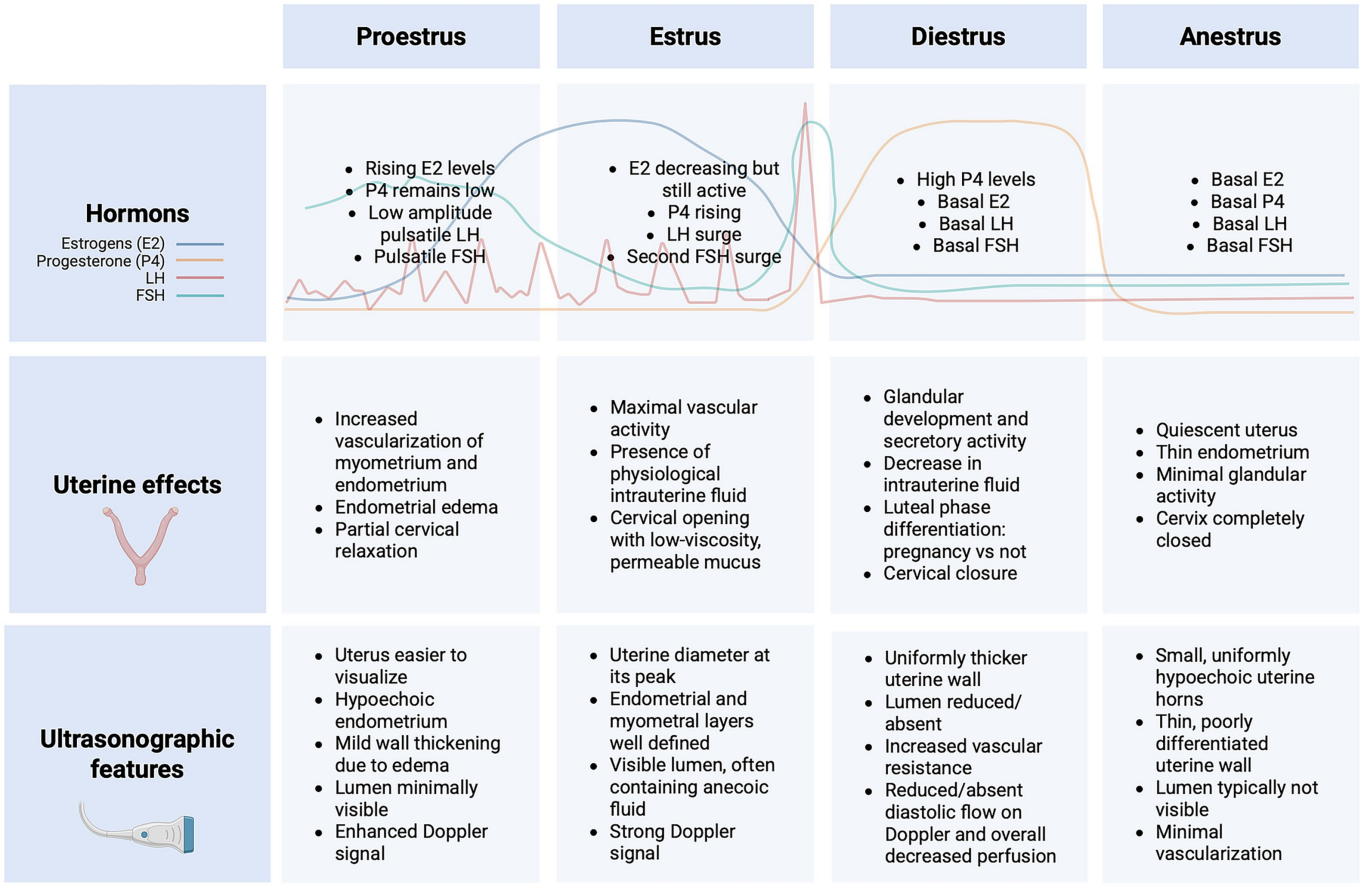
Schematic representation of the main hormones and corresponding uterine changes across the canine estrous cycle. Each phase is illustrated with its characteristic endocrine profile (estrogens, progesterone, LH, and FSH) and the associated structural modifications of the uterus, providing a visual framework that supports the interpretation of the ultrasonographic findings presented later in the manuscript. Created with BioRender.com under an institutional license (2025).

The Y-shaped uterus consists of two uterine horns, a small body, and a cervix, connecting cranially to the uterine tubes and caudally to the vagina. The uterus supports the development, implantation, and maintenance of the conceptus (8, 9). The uterine walls are composed of three main layers: the endometrium, the myometrium, and the perimetrium, corresponding to the tunica mucosa, muscularis and serosa, respectively. The endometrium contains glandular and epithelial components essential for implantation and placental development, while the myometrium consists of two smooth muscle layers responsible for uterine contractions during parturition (6). During pregnancy, the tunica mucosa undergoes hypertrophy, forming a placenta in conjunction with the fetal membranes. This structure is essential for nutrient and gas exchange between the dam and the developing fetus, acting as the primary source of nourishment and waste elimination (8, 10). The size and position of the uterus vary according to age, parity and reproductive status; in nulliparous 25-pound bitches, the uterine body measures approximatively 1.4 to 3 cm in length and 0.8 to 1 cm in diameter, with the horns averaging 10 to 14 cm in length and 0.5 to 1 cm in diameter (8). Anatomically, the uterus spans the pelvic and abdominal cavities, although most of its mass typically lies within the abdomen. In multiparous females, its increased size and elasticity allow the uterine body to extend cranially beyond the pelvic brim (8). As pregnancy advances, the gravid uterus can expand to occupy almost any region of the abdominal cavity. This displacement is influenced by the number and size of fetuses, and by the adaptability of the uterine ligaments. Although the suspensory and round ligaments restrict complete mobility, the uterine horns can still shift relative to one another to accommodate fetal growth. With increasing distension, the horns may flex inward and lie against the ventral abdominal wall, particularly in bitches carrying larger litters (8, 10). In addition to its dynamic role during gestation, the uterus also demonstrates cyclic changes influenced by hormonal fluctuations during the estrous cycle (7, 11–13). Figure 1 provides an integrated overview of the hormonal fluctuations and uterine anatomical changes characterizing each stage of the estrous cycle. By illustrating how variations in estrogen, progesterone, LH, and FSH drive modifications in uterine vascularization, endometrial architecture, cervical status, and the intrauterine environment, the figure also introduces the ultrasonographic patterns that are explored in detail in the following sections.

### 2.3 Cervix

The cervix, often forms the anatomical and functional gateway between the uterus and the external genitalia (8), protecting the (sterile) uterine environment from external contaminants and playing a key role in both reproductive and parturition processes (5, 9). In mammals, including humans, the cervix is typically described as having multiple layers: a mucosal lining that produces mucus to form a cervical plug during pregnancy, a muscular layer rich in dense connective tissue and smooth muscle fibers providing structural rigidity and elasticity, and an outer serosal layer (7). The mucus secreted by the cervical glands varies throughout the estrous cycle under hormonal influence. In dogs, direct studies on mucus properties are scarce; most descriptions derive from other species, where cyclic changes in viscosity, composition, and sperm permeability are well documented (14–16). Canine-specific evidence shows cyclic glandular changes and increased luminal hyaluronan during estrus (17–19), likely contributing to hormonally driven variations in mucus. During estrus, the mucus becomes less viscous to facilitate sperm transport, while during diestrus and pregnancy, it thickens to form a protective seal, preventing ascending infections (6). In a 25-pound dog, the cervix typically measures 1.5 to 2 cm in length with an approximate diameter of 0.8 cm (8). Its anatomical position is mostly pelvic, though orientation and shape may vary with reproductive status, becoming relaxed and open to allow sperm entry during estrus, while remaining tightly closed in anestrus (5, 11, 13, 19). During parturition, cervical ripening involves hormonal mediators (e.g., prostaglandins and relaxin) that remodel the extracellular matrix and reduce the tensile strength of collagen fibers, allowing dilation and softening to enable fetal passage (7). The role of the cervix extends beyond its mechanical functions: it also serves as a site for sperm reservoir formation, particularly in canines, where spermatozoa can be retained and gradually released to optimize fertilization success (10).

## 3 Technical considerations for reproductive ultrasonography in the bitch

**Figure 2:**
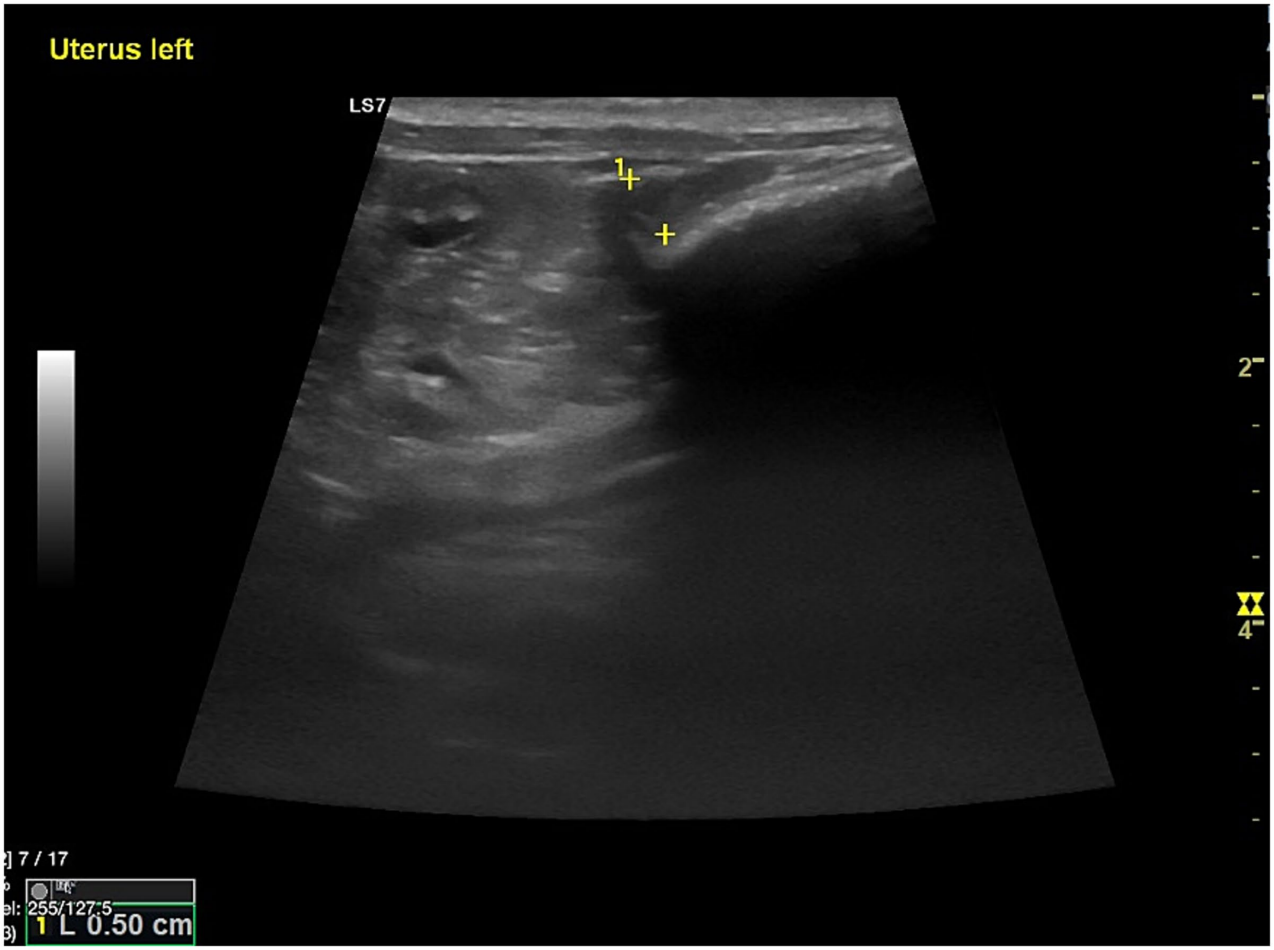
Sagittal ultrasonographic view of the uterus in a 4 kg bitch during anestrus. With the enhanced sensitivity of modern high-definition ultrasound systems, the uterine body and horns can be clearly distinguished even in small dogs and despite the naturally small uterine size characteristic of anestrus. In this example, the uterus (#1), identified between the caliper markers, shows a small diameter (approximately 0.5 cm), thin and uniform walls, and absence of a visible lumen, consistent with a hormonally inactive uterine state.

Optimal imaging of the canine reproductive tract requires a standardised approach adapted to each ultrasonographic modality. For transabdominal evaluation, a high-frequency transducer (7.5–12 MHz) offers the best balance between resolution and penetration, allowing detailed visualisation of both anatomical structures and vascular patterns (2). The same probe can be used in B-mode for morphological assessment and, when Doppler mode is activated, for functional evaluation of blood flow (20, 21). Patient positioning in dorsal recumbency generally provides optimal access to pelvic structures, although lateral or standing positions can be selected for specific clinical needs (22). Before scanning, the abdominal hair should be clipped from the xiphoid process to the caudal mammary glands, the skin wetted with alcohol, and acoustic gel applied to ensure optimal image quality (23). Fasting and a moderately full urinary bladder are recommended, as bladder distension acts as an acoustic window, improving uterine visualisation (23). In transverse planes, the bladder appears in the near field, the colon in the far field, and the uterine body in between (2). Rotating the transducer to a sagittal view reveals the uterine body and horns, which with modern high-resolution systems can be clearly identified without hormonal stimulation or pathological enlargement, as visually represented in Figure 2, which shows the uterine body and horns of a 4-kg bitch in anestrus measuring approximately 0.5 cm.

Intestinal gas or faecal material, however, can hinder pelvic inlet evaluation, particularly if the bladder is empty (2). For Doppler examinations, once the target vessels are identified in B-mode, color or power Doppler can be applied to assess perfusion patterns. Adjustments to gain and pulse repetition frequency are essential to minimise noise and aliasing artefacts (24). In spectral Doppler mode, a specific uterine vessel is isolated, and the sample volume is positioned centrally within the vessel to record velocity waveforms (25). This technique provides quantitative data by measuring blood flow velocities, including peak systolic velocity (PSV), end-diastolic velocity (EDV), and mean velocity (MV), which are used to derive indices such as the resistivity index (RI) and pulsatility index (PI), to provide information on vascular impedance and characterize the vascular supply of the uterus (25). When performing CEUS, the examination begins with a baseline B-mode and/or Doppler scan to define the region of interest (ROI). After intravenous administration of ultrasound contrast agents, dynamic vascular characteristics, including contrast presence or absence, wash-in (entry phase), wash-out (exit phase), peak enhancement, temporal behavior, vascular patterns, flow direction, and tissue perfusion are recorded in real time. Dedicated software can then quantify parameters such as peak intensity (PI), time to peak (TTP), and area under the curve (AUC), facilitating the differentiation between normal vascular physiology and pathological abnormalities (3). For elastography, the region of interest must first be clearly identified and centered using B-mode to ensure accurate anatomical localisation. Once defined, either manual compression (strain elastography) or acoustic impulses generated by the probe (shear-wave elastography) are applied to induce tissue deformation. The dedicated software then analyses the resulting displacement patterns, calculating either the elastographic index (strain) or the shear-wave propagation velocity (SWE), which are indicators of tissue stiffness and can be correlated with physiological or pathological changes (3, 26–28). 3D and 4D ultrasonography require accurate probe handling to capture high-quality volumetric datasets within a few seconds, minimising motion artefacts from respiration or fetal activity (29, 30). Acquired volumes can then be reconstructed for multiplanar analysis, surface rendering, and precise spatial measurements, making these modalities valuable for complex cases and research applications. Table 1 summarizes the main ultrasonographic modalities currently available to the evaluation of the canine uterus, along with their working principles, advantages, and limitations.

**Table 1:** Summary of main ultrasonographic techniques applied to the evaluation of the female canine reproductive tract, including principles of operation, advantages, limitations, and relevant references.

Technique	Principle of operation	Advantages	Limitations	References
B-mode	Emission and reception of ultrasound waves to create 2D grey-scale images	Widely available; fast, real-time, non-invasive; high spatial resolution for uterine/fetal structures; repeatable without radiation; detects small changes; combinable with other modalities	Limited specificity for similar echogenicity; no functional data; operator-dependent interpretation; image quality affected by gas, patient conformation, bladder filling	(2, 22, 23, 50, 56, 132–135)
Color, power and spectral Doppler	Analysis of frequency shifts in returning echoes caused by blood movement	Assesses uterine/placental vascularization with anatomical correlation; detects hemodynamic changes before structural ones; adaptable to clinical needs	Requires expertise; no specific reference values; reduced sensitivity for slow/deep flows	(20, 21, 24, 136–138)
CEUS	Use of echogenic microbubbles as contrast agents to assess tissue perfusion in real time	Real-time microvascular perfusion assessment; higher sensitivity than Doppler for low flow; quantitative/qualitative data; improves lesion characterization; portable compared to CT or MRI	Limited availability; needs contrast and training; short imaging window; no reference values; high cost	(139–144)
Ultrasound elastography	Measurement of tissue stiffness through induced deformation; strain: elastographic index, shear-wave: velocity	Adds biomechanical to morphological imaging; differentiates fibrotic, inflammatory and normal tissues; detects changes not seen on B-mode; complementary for staging/monitoring	Few reference data; steep learning curve; high equipment/software cost	(3, 4, 26–28, 145–147)
3D/4D ultrasonography	Volumetric acquisition with 3D reconstruction and real-time visualization	Comprehensive volumetric assessment; accurate measurement of volumes/relationships; retrospective dataset analysis; aids surgical/interventional planning; dynamic fetal/uterine evaluation	Longer acquisition/processing time; motion artefacts; high cost; limited validation in reproduction	(29, 30)

CT = computer tomography; MRI = magnetic resonance imaging.

## 4 Evaluation of reproductive structures

### 4.1 Introduction

**Figure 3:**
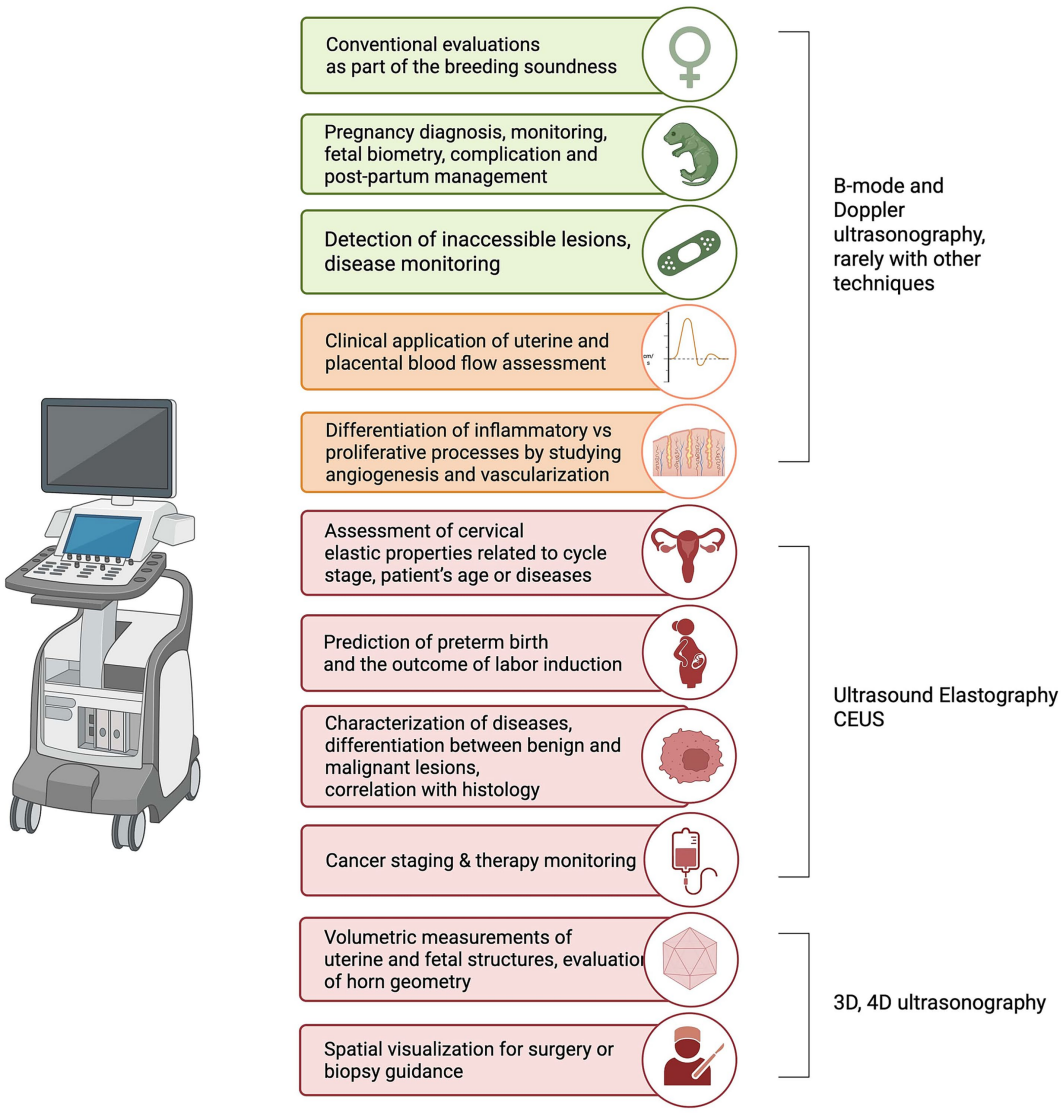
Overview of the principal clinical applications of ultrasonography for the evaluation of the canine uterus, its associated structures, and its contents. Applications are grouped by imaging modality and are color-coded to reflect their current level of validation and adoption in veterinary practice. In this color scale, green identifies techniques with robust evidence, high diagnostic reliability, and widespread integration into routine veterinary workflows. Orange denotes applications that are supported by promising data and moderate clinical adoption, but for which further validation or wider dissemination is still needed. Red highlights specialized or emerging uses that remain in early experimental stages or are largely derived from human reproductive imaging, with minimal or no current implementation in veterinary medicine. Created with BioRender.com under an institutional license (2025).

Before detailing the ultrasonographic appearance of the uterus, its associated structures and its contents, as well as the specific applications of different imaging modalities, Figure 3 provides an overview of the principal clinical uses of ultrasonography for uterine assessment in the bitch.

### 4.2 Uterine tube

#### 4.2.1 Normal findings

In normal conditions, the uterine tubes in bitches are typically not visible on routine ultrasonography due to their small size and similar echogenicity to surrounding tissues (31). They are thin, tubular structures that run from the ovaries to the uterine horns, and in the absence of significant fluid or pathological changes, they do not produce enough contrast with adjacent tissues to be easily differentiated.

#### 4.2.2 Abnormal findings

Pathological changes in the uterine tubes, such as inflammation or fluid accumulation, have not been extensively described in veterinary literature for dogs. However, in human medicine, these changes are well-documented and serve as a reference for understanding potential imaging findings. Salpingitis is associated with mild to moderate dilation. On ultrasonography, the tubes may appear slightly enlarged with a hypoechoic wall, and increased vascularity can be observed using Doppler ultrasonography, indicative of active inflammation (32, 33). When the tubes are filled with clear serous fluid, they become dilated and appear as tubular, anechoic fluid-filled structures. The degree of dilation depends on the amount of fluid present, with walls appearing thin but well-defined (34). In cases of pyosalpinx, the fluid may appear heterogeneous on ultrasonography due to the presence of pus, with hyperechoic or mixed echogenicity areas and irregular, thickened walls (32).

### 4.3 Non-pregnant uterus

#### 4.3.1 Normal findings

**Figure 4:**
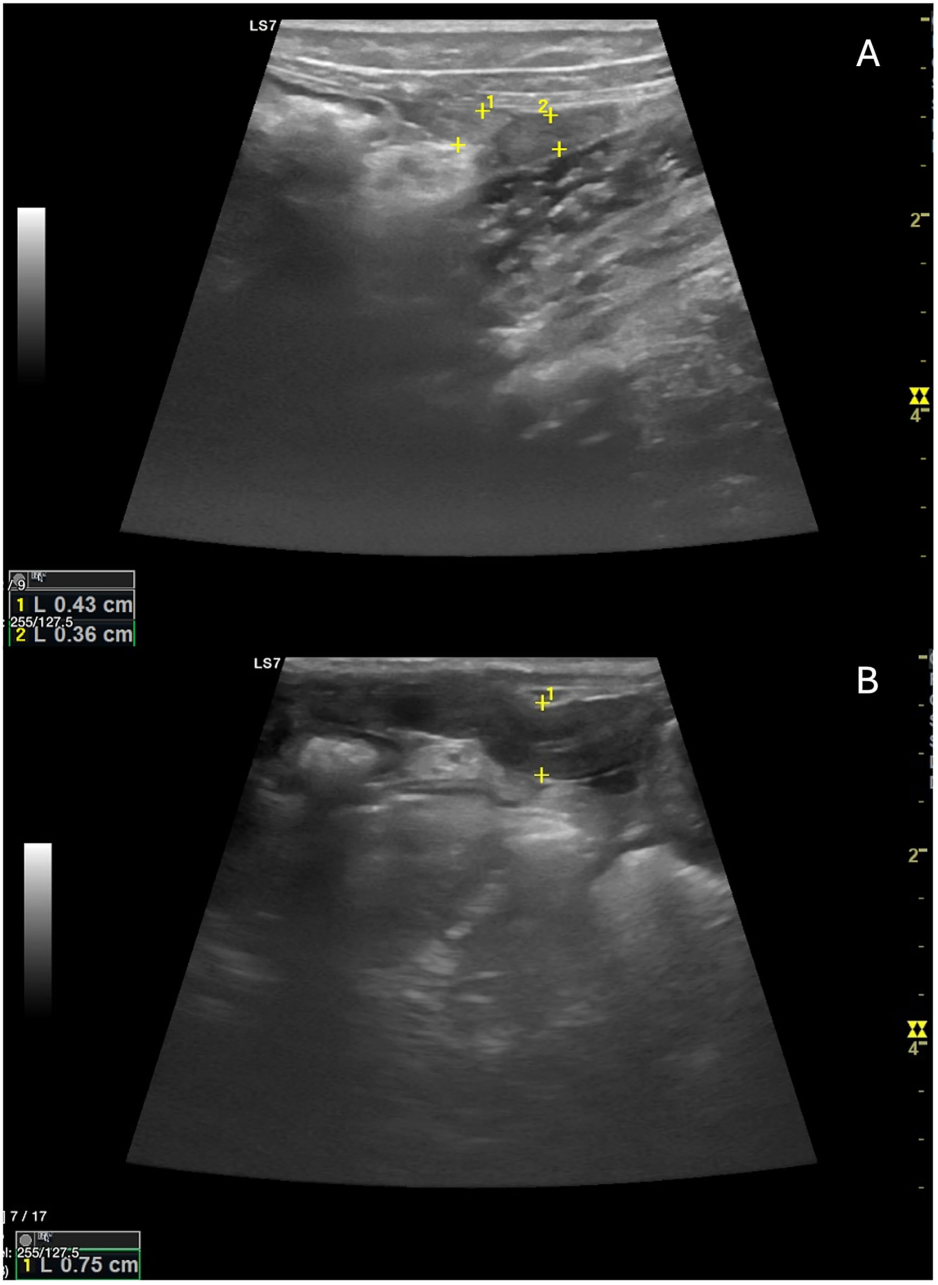
Ultrasonographic comparison of the uterus in the same bitch during anestrus and estrus. **(A)** In anestrus, the uterine horns (#1, 2), delimited by caliper markers (+), appear small with thin, homogeneous walls and no visible luminal cavity, reflecting the quiescent physiological condition of this phase. **(B)** During estrus, the uterine horn (#1), highlighted by the caliper markers (+), displays increased diameter and wall thickness. The uterine lumen becomes visible together with more evident wall layering, consistent with estrogen-induced vascular and endometrial activation.

The ultrasonographic and hemodynamic appearance of the uterus in dogs varies significantly throughout the estrous cycle, influenced by hormonal changes and individual factors such as breed, body size, and reproductive history (22). During anestrus, the uterus appears small and uniformly hypoechoic on grey-scale ultrasonography, with a thin, poorly differentiated wall and an inconspicuous lumen (Figure 4A) (23). Correspondingly, Doppler studies show minimal vascularization, with high PI and RI values, indicating reduced perfusion (35). As proestrus begins, rising levels lead to uterine wall thickening due to edema and increased vascularization. On B-mode, the endometrium becomes more prominent and may contain small amounts of anechoic fluid, giving the uterus a coiled or serpentine appearance (23). Doppler reveals elevated PI due to increased vascular impedance and lower RI, indicating a shift toward active endometrial perfusion (25). During estrus, uterine diameter reaches its peak, and the myometrial and endometrial layers become more defined (Figure 4B). Anechoic intrauterine fluid is often more evident, especially in mated bitches (2). Uterine horns are more easily visualized due to their distension.

Doppler parameters show decreased PI compared to proestrus, reflecting reduced impedance, while RI remains stable, supporting ovulation and fertilization (36). In diestrus, under the influence of progesterone, the uterus maintains a thick and more homogeneous appearance on ultrasonography. Although the endometrial glandular activity persists, echogenicity begins to increase and fluid content decreases as hormonal support wanes (25). Vascular resistance increases again, with both PI and RI rising, and Doppler waveforms show reduced diastolic flow, consistent with reduced perfusion (35). Notably, studies have shown a transient dip in uterine blood flow two days prior to ovulation, followed by a gradual post-ovulatory increase, possibly associated with the vascular shift from follicular to luteal dominance (36).

#### 4.3.2 Abnormal findings

##### 4.3.2.1 Endometritis, cystic endometrial hyperplasia (CEH), and uterine fluid accumulations (pyometra, hydrometra, mucometra, and haemometra)

These conditions represent the most frequent uterine disorders encountered in clinical practice, and although related, they differ in pathogenesis, severity, and ultrasonographic presentation. Endometritis, cystic endometrial hyperplasia (CEH), and uterine fluid accumulations represent a continuum of hormonally influenced but distinct pathological conditions, often interrelated and predominantly occurring during diestrus. Elevated progesterone levels during this phase stimulate endometrial gland proliferation while suppressing immune defense, creating a favorable environment for fluid accumulation and secondary infection (37).

*Endometritis* is the inflammation of the endometrial lining, commonly caused by bacterial infections ascending from the lower reproductive tract, particularly during phases where the cervix remains open, such as mating or insemination (38). Post-mating endometritis, in particular, is triggered by bacterial colonization due to cervix dilation during copulation, which facilitates the entry of bacteria into the uterus (39). Inflammatory cells, notably polymorphonuclear neutrophils (PMNs), flood the uterine lumen in response to bacterial invasion, and this inflammation, if unresolved, can reduce pregnancy rates and litter size (40). This condition involved transient vasodilatation and increased perfusion to facilitate sperm transport and implantation. This vascular response is detectable with Doppler ultrasonography as a temporary decrease in RI and PI, accompanied by increased PSV and EDV (39). If persistent, the inflammatory process can cause structural damage to the endometrium, increasing the risk of infertility or further complications such as CEH (38, 41, 42). B-mode ultrasonography may reveal uterine wall thickening with increased echogenicity caused by edema and inflammation, sometimes accompanied by small amounts of luminal fluid ranging from anechoic to hypoechoic depending on its composition and the severity of the condition (2, 23). Despite these potential findings, the sonographic appearance of endometritis often overlaps with that of CEH or early pyometra, and mild or subclinical forms may remain undetected. Consequently, additional diagnostic tools such as cytology and histology are required for a definitive diagnosis (23, 39, 40). Recent advances have introduced computer-assisted ultrasonography to reduce the subjectivity of visual assessment. Quantitative analysis of endometrial echogenicity and heterogeneity revealed that pyometra significantly increases both parameters, whereas CEH and endometritis do not significantly differ from normal uteri (43). Digital analysis of intraluminal contents also allows differentiation between purulent, mucous, and serous fluids, with purulent content being more echogenic and heterogeneous due to higher cellularity and bacterial load (44). While these approaches are promising, their current diagnostic utility for endometritis remains limited, and their main value lies in research and the potential future integration with automated or machine-learning-based image interpretation. Doppler parameters provide functional information on uterine perfusion. Recent studies have demonstrated that uterine artery velocimetry can differentiate endometritis from both CEH and normal uteri. Specifically, endometritis is associated with an intermediate RI, lower than in healthy bitches but higher than in CEH, while EDV is reduced compared with CEH (45). These hemodynamic changes reflect vascular congestion and neutrophilic infiltration, underscoring the contribution of angiogenesis and inflammation to uterine perfusion (45).

**Figure 5:**
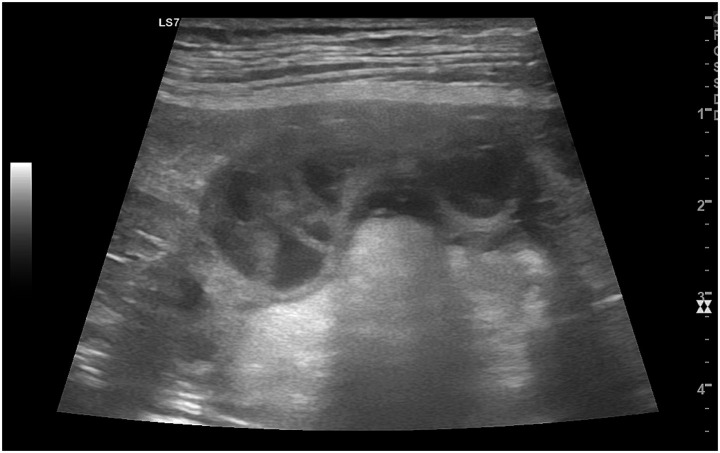
B-mode ultrasonographic image of the uterus in a bitch affected by cystic endometrial hyperplasia (CEH). The uterus is clearly identifiable as an enlarged tubular structure with an irregularly thickened wall. Within the endometrium, multiple round to oval anechoic to mildly hypoechoic cystic structures of variable size are visible, corresponding to glandular cystic dilatation. The normal layered appearance of the uterine wall is partially lost, with distortion of the endometrial architecture, findings that are characteristic of cystic endometrial hyperplasia.

*CEH* is a chronic, hormone-mediated condition of the endometrium, where prolonged exposure to progesterone results in abnormal thickening of the endometrial lining, leading to the formation of cysts (46). Concomitant estrogens also play a relevant role by priming the endometrium and upregulating progesterone receptors; this is particularly evident in severe cases often associated with ovarian cysts or persistent follicles producing estrogen (47–49). The endometrial cysts are filled with secretory material and can predispose the uterus to secondary bacterial infections, often progressing to pyometra (CEH–pyometra syndrome), and although CEH often co-occurs with endometritis, it can develop independently (37, 46). B-mode ultrasonography typically shows a thickened, echogenic endometrium with multiple small anechoic cysts (Figure 5) (2, 50).

CEUS studies demonstrated rapid, homogeneous enhancement of hyperplastic tissue with absent perfusion in cystic areas, highlighting viable versus non-vascularized regions and correlating with histopathology (51).

**Figure 6:**
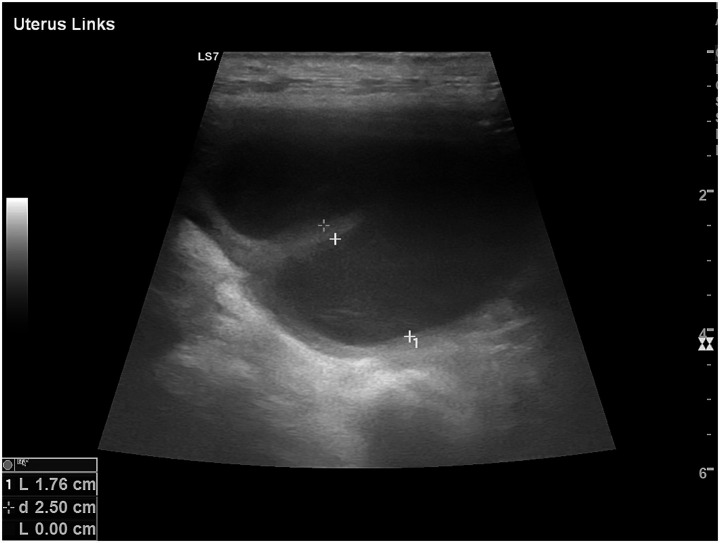
Ultrasonographic appearance of the uterus in a bitch with pyometra. The uterine horn (#1), outlined by caliper markers (+), is markedly enlarged and filled with heterogeneous, debris-containing echogenic fluid, consistent with purulent intrauterine content. The uterine wall appears thickened and irregular, reflecting severe inflammatory changes.

*Pyometra* is a life-threatening condition characterized by the accumulation of purulent material within the uterus often developing on a CEH background but potentially arising independently (37, 46). The condition is usually associated with elevated progesterone levels during diestrus, leading to reduced uterine contractility and suppression of the immune response, creating an ideal environment for bacterial growth (37). In contrast, hydrometra, mucometra, and haemometra represent non-infectious forms of intrauterine fluid accumulation, involving serous, mucous, or blood-tinged fluid, respectively (42). Pyometra typically presents with echogenic, debris-filled fluid (Figure 6), while hydrometra and mucometra are usually anechoic or hypoechoic (2, 23, 50). With pyometra, the enlargement is usually symmetric, but segmental or focal changes can occur, and the condition can also occur in one uterine horn with a viable pregnancy in the other (2). Haemometra may contain hypoechoic to echogenic clots, making its appearance more variable (52). However, ultrasonography alone is insufficient for distinguishing between these conditions, and the echotexture of the fluid is not always a reliable indicator of the specific condition. Definitive diagnosis often requires other findings to confirm infection and guide appropriate treatment (37).

Doppler ultrasonography serves as a crucial diagnostic tool to evaluate these changes, offering insights into the severity and progression of uterine pathology. PI and RI were observed to decrease progressively from the uterus of healthy bitches, through those with CEH, to those with pyometra. Healthy, non-pregnant bitches exhibited the highest PI and RI values, reflecting lower uterine perfusion under normal physiological conditions (35, 53, 54). In contrast, the reduced PI and RI in CEH suggest moderate vascular changes, while the pronounced decline in these indices in pyometra indicates significant vasodilation and increased blood flow associated with severe inflammation (46, 53). Similarly, the PSV and EDV showed a marked increase across the same conditions. In healthy, non-pregnant bitches, these values were lowest, consistent with the basal metabolic demands of the uterus (53). In CEH, the moderate rise in PSV and EDV reflects the vascular adaptation to the proliferative endometrium, and the dramatic elevation in both velocities in pyometra underscores the heightened uterine perfusion required to meet the demands of an inflamed and infected uterine environment (25, 53). This increased blood flow is likely driven by local vasodilators, such as prostaglandin E and nitric oxide, which reduce vascular resistance and promote angiogenesis, further emphasizing the hemodynamic impact of pyometra (53).

##### 4.3.2.2 Uterine tumors

Uterine tumors in dogs, although uncommon, present with a wide spectrum of biological behaviors and ultrasonographic appearances, ranging from benign lesions such as endometrial polyps and leiomyomas to malignant forms including fibroleiomyomas, leiomyosarcomas, and adenocarcinomas (2, 55). Clinical presentations are variable, from asymptomatic cases to signs such as vaginal discharge, uterine enlargement, and infertility. On grey-scale ultrasonography, these tumors exhibit different appearances depending on their type, structure, and stage of development. Leiomyomas, the most common benign tumor in middle-aged to older dogs, are characterized by slow growth, non-invasive behavior, and a lack of metastasis (31). Fibroleiomyomas and leiomyosarcomas, although different in behavior, share a similar ultrasonographic appearance, typically presenting as solid, well-defined, hypoechoic masses, often leading to uterine enlargement (52, 56). However, leiomyomas and leiomyosarcomas can also present with anechoic ischemic cavities within the mass, giving a mixed to cystic appearance and hyperechoic foci may be observed within these tumors, which could indicate calcification, fibrosis, or metaplasia (55). This discrepancy suggests that the ultrasonographic presentation may vary depending on the progression of the tumor or its internal structure. Poorly differentiated sarcomas have been typically described as solid masses, while adenocarcinomas, though rare, are sometimes observed as mixed echogenic masses with a combination of solid regions, hyperechoic areas, and cystic components (55). Their heterogeneous nature can make them difficult to differentiate from other uterine masses, though their more aggressive behavior often leads to local invasion and metastasis (55). Endometrial polyps are tumour-like lesions reported frequently in domestic carnivores (57). Polyps appear as well-demarcated, endoluminal masses, which may be solid or contain multiple anechoic cystic glands. Grossly, they range in size from 5 to 25 cm in diameter and may present as single, sessile, or pedunculated structures, often associated with cystic endometrial hyperplasia (57). Microscopically, polyps are fibroglandular, with stroma that may exhibit hemorrhagic areas infiltrated by inflammatory cells. In some cases, features such as stromal smooth muscle and epithelial squamous metaplasia have been observed, potentially indicative of preneoplastic changes (57). Doppler ultrasonography contributes additional diagnostic value by characterizing tumoral vascularity, which often differs markedly from that of healthy tissue. Neoplastic tissues commonly display increased and irregular vascular flow, attributed to chaotic angiogenesis, altered vessel architecture, and changes in intra- and extravascular pressure, as extensively described in human oncology (58). While these mechanisms have not been fully characterized in canine uterine neoplasms, they provide a useful framework for understanding similar pathological vascular changes in dogs. Although specific Doppler indices are not reported for canine uterine tumors, general trends include increased perfusion: uterine neoplasms such as leiomyomas and adenocarcinomas display heterogeneous echotexture with regions of increased vascular flow on color Doppler, reflecting their metabolic demand and angiogenic activity (55). From a previous study, malignant tumors seem characterized by chaotic and irregular vascular networks, with reduced RI and PI values reflecting the low resistance of tumor vasculature, and exhibit non-uniform flow distribution and abnormal vessel organization, distinguishing them from the more structured and predictable vascular patterns of benign lesions (59). Notably, even benign tumors such as endometrial polyps may demonstrate elevated vascularity, sometimes mimicking malignant profiles, thus complicating the interpretation of Doppler findings (60). Consequently, while Doppler aids in identifying vascular abnormalities suggestive of neoplasia, histopathological confirmation remains essential for accurate tumor classification and malignancy grading.

##### 4.3.2.3 Pseudo-placentational endometrial hyperplasia (PEH)

**Figure 7:**
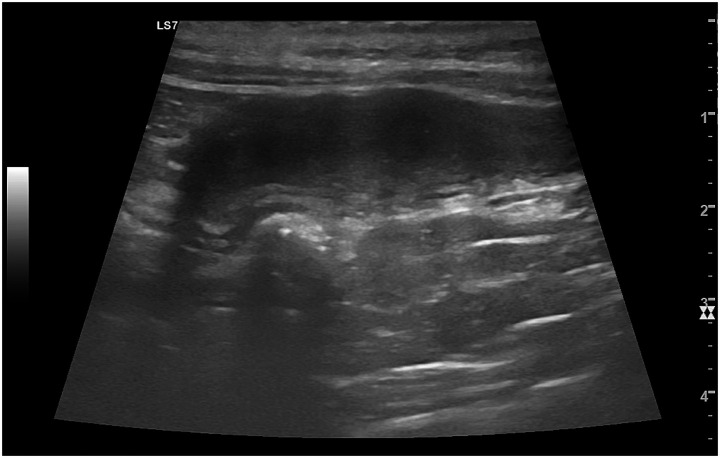
B-mode ultrasonographic image of the uterus in a bitch with pseudo-placentational endometrial hyperplasia (PEH). The uterus appears enlarged and contains a focal, well-demarcated intramural thickening characterized by predominantly hyperechoic tissue. This focal lesion causes localized distortion of the uterine wall and produces an appearance that may mimic placental remnants or other space-occupying uterine masses. The diagnosis of PEH was subsequently confirmed by gross examination and histopathology, which demonstrated a characteristic placental-like architecture.

Pseudo-placentational endometrial hyperplasia is a rare, non-inflammatory proliferative lesion of the canine uterus, characterized by the formation of intramural nodules that resemble placental tissue. This condition is distinct from CEH and involves well-organized tissue architecture that mimics the layers of the maternal placenta, including a glandular basal layer, a connective band, and a spongy labyrinth layer (61). Ultrasonographically, PEH manifests as focal uterine swellings with hyperechoic tissue (Figure 7) that may be mistaken for placental remnants or other uterine pathologies, such as neoplasia (31, 61). However, histopathological confirmation is required for a definitive diagnosis, as ultrasonography alone cannot reliably distinguish PEH from other conditions.

##### 4.3.2.4 Uterine torsion

Uterine torsion is an uncommon yet life-threatening condition in which the uterus twists along its axis (62). Although more frequently associated with pregnancy, torsion can also occur in non-pregnant bitches (63), particularly in the presence of underlying uterine pathologies (64) or inflammatory endometrial polyps (65). On grey-scale ultrasonography, the condition typically appears as a severely distended uterus with marked wall thickening, sometimes accompanied by abdominal effusion and signs suggestive of peritonitis (31, 50). Additional sonographic signs may include displacement of adjacent organs and asymmetry between uterine horns, although these features are nonspecific. Doppler ultrasonography plays a key role in confirming vascular compromise by revealing absent or severely diminished blood flow in the affected uterine artery (31). Although direct canine studies are limited, bovine models have shown that uterine torsion causes elevated PI and RI, reflecting arterial and venous outflow obstruction due to mechanical twisting (66, 67). Doppler waveforms often reveal an absence of early diastolic flow and persistent notches, reflecting severe vascular compromise that can result in poor uterine perfusion, ischemia, or tissue necrosis (66). Furthermore, the degree and severity of torsion can be assessed through Doppler parameters, as greater alterations in blood flow indices are associated with more advanced or prolonged torsion (68).

##### 4.3.2.5 Uterine stump pathologies

After ovariohysterectomy, the remaining uterine stump may develop pathologies such as granulomas, stump pyometra, hematomas, or tumors (2, 23, 69). Granulomas occur secondary to foreign bodies, reactive suture material, or focal infection and can cause chronic infections in the ovariohysterectomized bitch. Granulomas appear as hypoechoic, well-defined masses at the site of the stump, and may be associated with inflammation or infection (2, 23). Stump pyometra can develop following progesterone stimulation from residual ovarian tissue, leading to glandular stimulation and secretory activity in the uterine stump (70). It can also occur after estrogen exposure, which has been shown to influence uterine stump morphology in ovariohysterectomized dogs (71). This condition is often identified using ultrasonography, which can reveal fluid distension in the uterine remnant, a characteristic feature of stump pyometra (52). Hematomas, appearing as echogenic masses, often with fluid–fluid levels, are typically a postoperative complication (2, 52).

##### 4.3.2.6 Congenital defects

Congenital defects, such as the absence of one or both uterine horns (uterine agenesis), oviductal aplasia or segmented uterus, are rare but can be detected through imaging. These abnormalities present as an absence or hypoplasia of the affected reproductive structures. By ultrasonography, the absence of a uterine horn or oviduct may be confirmed by visualizing only one horn, or by the lack of normal tubular structures in the expected anatomical location (72).

### 4.4 Pregnant uterus

#### 4.4.1 Normal findings

##### 4.4.1.1 General consideration for imaging the pregnant uterus

Ultrasonography is essential for monitoring canine pregnancy, offering detailed insights into gestational age, fetal viability, organ development, and placental function. Its real-time, non-invasive nature allows for repeated assessments throughout gestation, supporting optimal management particularly in high-risk situations such as singleton pregnancies or oversized litters (73–75). While grey-scale ultrasonography remains the primary modality for anatomical evaluation, Doppler ultrasonography adds valuable functional information by assessing maternal and fetal blood flow. However, both techniques present specific challenges. In grey-scale, accurate fetal counting becomes increasingly difficult as gestation advances: overlapping structures, displacement of uterine horns by abdominal organs, and anatomical variations—especially in large litters or near the uterine body-bifurcation—can lead to under- or overestimation (23, 69, 76). For this reason, radiographic confirmation closer to term is often required for definitive fetal counts (2, 23, 76). Doppler ultrasonography, though extremely informative, requires technical precision and operator experience. Insonation angles greater than 65 degrees should be avoided as they can result in inaccurate data, while angles between 0 and 20 degrees are ideal for precise assessment (77, 78). The technique is time-intensive and demands significant experience to obtain reproducible results, especially in complex cases such as those involving multiple fetuses. The ability to consistently evaluate the same vessel in the same location during serial examinations is another challenge, particularly in canine pregnancies. The presence of multiple fetuses and the shifting positions of the uterine horns throughout gestation can complicate longitudinal assessments of individual fetal or maternal vessels. As a result, unless a fetus or fetuses are identified as abnormal based on developmental markers, following changes in blood flow for a specific fetus over time can be difficult (76). Additionally, while RI and PI measurements provide valuable information about uterine and umbilical artery blood flow, alterations in diastolic flow and the presence of a diastolic notch must be interpreted carefully in relation to gestational age and fetal well-being (78, 79). These factors are crucial for evaluating fetal readiness for birth, yet variability in vascular measurements and fetal positioning can limit their consistency.

##### 4.4.1.2 Pregnancy diagnosis and progression

Canine pregnancy can be detected via ultrasonography as early as 19 days after the LH surge (76), when spherical gestational sacs become visible, measuring approximately 1 mm in diameter (75, 76). By day 23, the embryo, accompanied by detectable cardiac activity, appears as an elongated structure attached to the uterine wall (75, 76, 80). Around day 26–27 post-LH surge, the canine placenta becomes ultrasonographically visible, gradually developing its characteristic zonary structure. By days 29–31, the placenta exhibits a distinct zonary appearance with curled edges clearly detectable on ultrasonography (76). Between days 29 and 33, the stomach and urinary bladder emerge as identifiable anechoic areas, and skeletal mineralization starts, presenting as hyperechoic structures (76). By days 35 to 38, the thoracic and abdominal cavities are clearly differentiated, with the lungs appearing hyperechoic compared to the liver, which remains hypoechoic relative to other abdominal organs (76). Further organ development includes the kidneys, which become visible between days 39 and 47, and the intestines, which can be identified as early as day 39, with peristaltic movements becoming consistently visible between days 62 and 64. Although peristaltic movements of the bowel mark the completion of fetal organogenesis, they should not be used as the sole parameter for determining fetal maturity or readiness for birth (76, 80, 81). Fetal sex determination is also feasible by ultrasonography. Around day 45 post-ovulation, external genitalia differentiate sufficiently to allow identification, provided imaging conditions and operator expertise are adequate (82). Ultrasonographic measurements provide critical data for estimating both gestational age and the expected date of parturition. Gestational age is conventionally calculated from the LH peak, considered day 0 of gestation, whereas in field practice ovulation, which occurs approximately 1–2 days later (83–85), is often used as a practical reference. During early gestation, the inner chorionic cavity diameter (ICC) is the most reliable parameter for estimating gestational age, particularly before day 35, offering relatively accurate predictions when species- and size-specific formulas are applied (76, 86). After day 37, the biparietal diameter (BPD) becomes the primary metric for estimating gestational age and predicting parturition. BPD measurements, taken as the widest distance between the fetal parietal bones, are most accurate during the second half of pregnancy and show a strong correlation with GA, offering robust precision across various breeds (76, 86, 87). Other ultrasonographic parameters, such as the outer chorionic cavity (OCC) diameter, fetal body diameter (BD), femur length (FL), and placental thickness, have been investigated as complementary markers. OCC is most useful during mid-gestation when BPD measurements are suboptimal, whereas BD and FL can support gestational age estimation in cases where fetal cranial landmarks are difficult to visualize, such as in large breeds or with suboptimal fetal positioning (76). Placental thickness has also shown a positive correlation with gestational age and can provide satisfactory accuracy, although earlier studies and reviews emphasized that ICC and BPD remain the most reliable and widely accepted parameters for clinical prediction (88). Recent investigations have also explored the potential of 3D ultrasonography to improve early gestational age prediction by measuring the ICC volume. However, these studies demonstrated that 3D ICC volume does not provide a significant advantage over conventional 2D diameter or length measurements due to the complexity of acquisition and the presence of motion artifacts, reaffirming the practicality and accuracy of the traditional approach (30). From a hemodynamic perspective, Doppler ultrasonography reveals progressive adaptations in maternal and fetal perfusion. In early pregnancy, uterine artery flow is characterized by high resistance, reflected in elevated RI and PI values, and relatively low PSV and EDV (76). These values begin to change markedly as pregnancy progresses. In mid to late gestation, there is a significant reduction in RI and PI, reflecting a decline in vascular resistance as the uteroplacental circulation becomes fully established (76). These changes are likely associated with the growing fetoplacental unit and the physiological adaptations of the maternal vasculature to support pregnancy (78, 79, 89). Perfusion in the umbilical artery begins to increase by week five of gestation, driven by the enlarging uterus and the increasing metabolic demands of the fetuses. RI and PI in the umbilical artery decrease significantly from week five to week eight, reflecting enhanced blood flow to meet these demands (78, 90, 91). In small breed bitches, RI values for the umbilical artery range from 0.64–0.79 at 30 days of gestation and decrease to 0.52–0.66 by 60 days, indicating consistent vascular adaptation (92). Peak systolic velocity and EDV increase substantially during this period, indicating enhanced uterine blood flow to meet the metabolic and oxygen demands of the growing fetus (42, 76, 90). This hemodynamic shift is driven by estrogen-mediated vasodilation and structural remodeling of the uterine arteries (42). Moreover, critical changes in Doppler waveforms occur as parturition approaches. As parturition approaches, notable Doppler waveform changes occur. Diastolic flow appears in the umbilical artery around 21 days before delivery, and the diastolic notch disappears 16–21 days pre-partum—key markers of placental vascular maturity (79).

##### 4.4.1.3 Fetal assessment and readiness for birth

**Figure 8:**
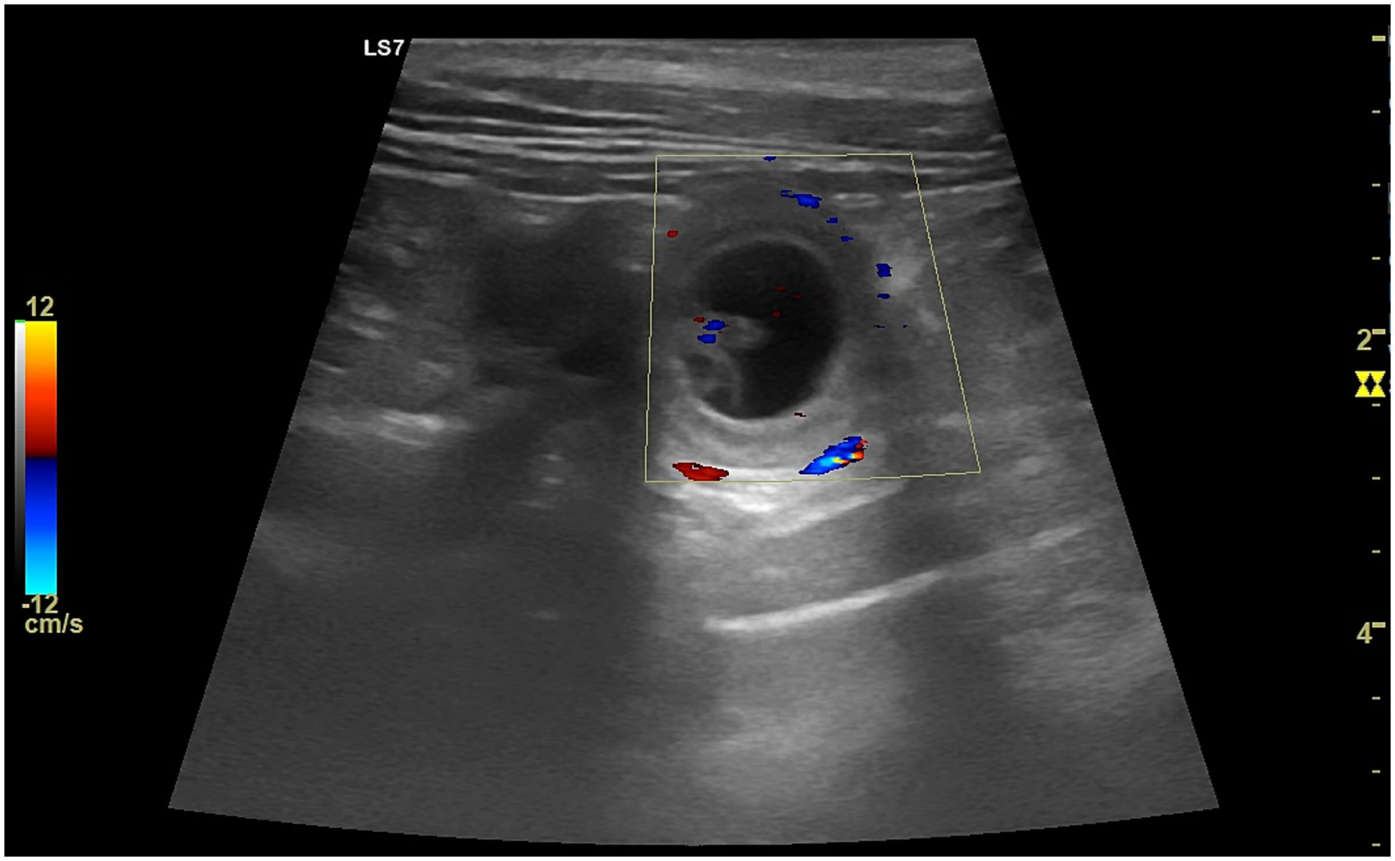
Color Doppler ultrasonographic image of an early canine gestational sac. The gestational sac is visible as a well-defined, round anechoic structure containing an embryo. Color Doppler examination highlights the presence of blood flow within the embryo, visible as vascular signals, indicating embryonic cardiac activity and confirming viability. The signal is also visible adjacent to the embryo and corresponds to developing embryonic vessels.

Ultrasonographic evaluation of the fetus provides essential information on development, well-being, and maturity during canine pregnancy (Figure 8).

From the fifth week post-insemination, blood flow in the fetal aorta is regularly detectable (89). Early in gestation, SPV in the fetal aorta increases significantly until week eight, after which it stabilizes in the last two weeks before parturition (89, 93). Diastolic flow, initially absent, begins to appear by week five and becomes consistent by week eight, indicating progressive vascular development (89). RI and PI decrease progressively during gestation, reflecting reduced vascular resistance as blood flow adapts to fetal growth and metabolic demands (76, 89, 93). Blood flow in the fetal common carotid artery is typically visible from week six, showing a biphasic waveform with sharp systolic peaks and flat diastolic velocities. Systolic peak velocity and EDV increase significantly from week six to week nine, while RI shows a slight decrease (89). The PI remains relatively stable, with no significant weekly changes. Moderate correlations between umbilical and aortic Doppler parameters, especially EDV, highlight the interplay between fetal and placental circulation (78, 89). Though less commonly assessed, the caudal vena cava also contributes useful data, particularly regarding fetal cardiac function (89). Venous flow patterns, when evaluated alongside arterial signals, offer a more comprehensive picture of fetal hemodynamics (78). Fetal heart rate remains consistently high throughout gestation, typically ranging between 200 and 220 beats per minute (bpm) in healthy pregnancies, reflecting the active metabolic state of the developing fetus (76, 94). In pregnant bitches, CEUS has been shown to be a safe and effective tool for evaluating maternal-fetal vascularization, as contrast agents do not cross the placental barrier, ensuring fetal safety during imaging (95). Qualitative and quantitative evaluations of uterine and placental vessels at different gestational stages have been performed, revealing homogenous contrast distribution from the uterine artery to placental vessels during the wash-in phase, followed by gradual wash-out (95). Quantitative parameters such as AUC and PI remained consistent across proximal and distal placental regions, while AUC in the uterine artery decreased from day 23 to day 30, likely reflecting structural changes in the uterine vasculature (95). Other studies explored CEUS parameters in brachycephalic bitches with normal and abnormal pregnancies. In healthy pregnancies, placental perfusion remained consistent, with homogenous enhancement during the wash-in and wash-out phases (96). Conversely, CEUS identified heterogeneous perfusion, delayed TTP, and reduced contrast intensity in cases of gestational abnormalities like anasarca and hydrocephalus, highlighting CEUS’s diagnostic accuracy (75%) in identifying placental dysfunction that may not be evident with B-mode or Doppler ultrasonography alone (96). Elastography has been used to assess the mechanical properties of fetal organs in the final days of gestation. Acoustic Radiation Force Impulse measurements in the fetal lungs and liver show stable shear wave velocities (SWV), suggesting consistent tissue stiffness as these organs mature. Lung echotexture, measured via numerical pixel values, correlates with the progression through canalicular and saccular phases, reflecting advanced pulmonary development. However, no direct correlation was found between SWV and echotextural metrics (97).

##### 4.4.1.4 Selective death

Ultrasound-guided selective fetal death is a precise and minimally invasive technique that enables the targeted reduction of specific fetuses while preserving the overall pregnancy (98). The transabdominal intracardiac injection of an agent to induce fetal cardiac arrest has been described as a method that minimizes side effects and ensures the viability of the remaining fetuses, making it particularly suitable for managing multifetal pregnancies or addressing medical indications during gestation (98).

##### 4.4.1.5 Post-partum/uterine involution

**Figure 9:**
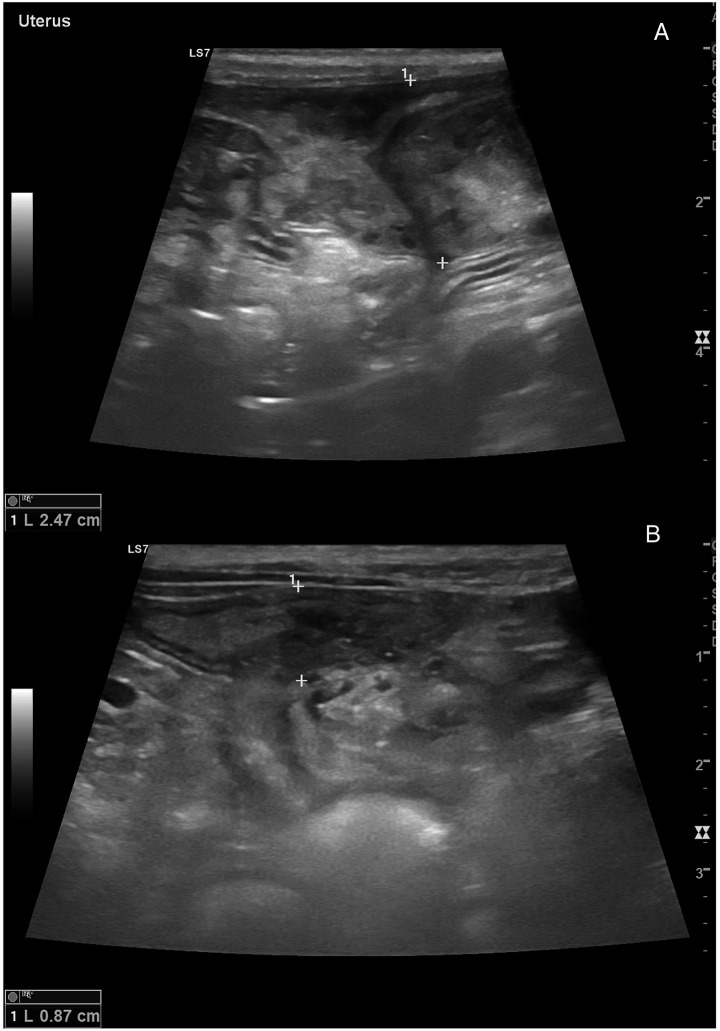
Ultrasonographic appearance of the uterus of a medium-sized bitch during the early postpartum period. **(A)** Immediately postpartum, the uterine horn (#1), identified between caliper markers (+), is enlarged with thickened, edematous walls and mixed-echogenic luminal content, consistent with normal early uterine involution. **(B)** Ten days postpartum, the same uterine horn (#1) shows a marked reduction in diameter and wall thickness, with minimal residual luminal content, indicating progression of physiological involution.

Postpartum uterine involution is a critical physiological process characterized by the gradual regression of the uterus to its pre-gestational state. B-mode ultrasonography could provide comprehensive insights into the morphological changes of the uterus during postpartum involution in bitches. In the early postpartum period (1–4 days), the uterine horns appear dilated, edematous, and enlarged, with placentation sites measuring between 1.5–3 cm (99–101) in diameter, as visually represented in Figures 9A,B. Different ultrasonographic aspects of the uterus of a medium-sized bitch can be observed during the early postpartum period. Interplacental sites range from 1.0–1.5 cm, and luminal contents display mixed echogenicity due to blood clots, mucus, and remnants of fetal and maternal membranes (100). The uterine wall during this period is thick, irregular, and moderately echogenic, often with visible blood vascularization (100).

By the fourth week postpartum, the uterine horns contract significantly, with placentation sites becoming nodular and pale, reducing in size to 1.0–1.4 cm, while interplacental sites measure 0.6–0.9 cm. The uterine lumen, initially visible due to the presence of anechoic fluid, becomes less detectable as the involution progresses and the uterine layers homogenize (36, 99). By the ninth week, placental sites form a narrow brown band, and complete involution, marked by uniform uterine dimensions and echogenicity, is typically achieved around the twelfth week postpartum (2, 36, 99, 101). Quantitative ultrasonography revealed significant differences in uterine involution between dogs undergoing natural delivery and those subjected to cesarean section. During the first 7 days postpartum, uterine diameter was significantly larger in the cesarean group compared to the natural delivery group (102). Uterine involution occurred more rapidly in dogs that delivered naturally, likely due to enhanced physiological processes such as the action of prostaglandins and oxytocin, which stimulate effective uterine contractions; in contrast, cesarean delivery may impede uterine regression, possibly due to surgical manipulation and associated trauma (102). Doppler ultrasonography reveals progressive vascular regression throughout postpartum. In the first week, placental sites remain hypervascularized, and turbulent flow can be visualized, reflecting active remodeling (99, 101). Uterine artery blood flow decreases steadily; PI and RI gradually increase, reflecting reduced uterine perfusion as vascular resistance rises, coinciding with the regression of gestational vasculature (21, 36). Peak systolic and end-diastolic velocity, which were elevated during pregnancy to meet the demands of fetal growth, progressively decline as parturition is finished (36). By the fourth week, a significant reduction in uterine diameter and vascular activity is observed, correlating with the histological resolution of necrotic tissue and the regeneration of endometrial glands. Placental sites, previously hypervascularized, exhibit diminished blood flow as the connective tissue remodels into a more fibrotic state (36, 99). From week seven to nine, the uterine horns show minimal luminal diameter, and Doppler assessments reveal low vascular activity, consistent with near-complete vascular regression. The absence of turbulent flow in the uterine vessels by this stage signifies the completion of vascular remodeling and the restoration of a non-pregnant hemodynamic state (36, 103).

Regarding elastography, ARFI has been applied to monitor uterine stiffness during the early postpartum period in dogs, highlighting key differences between natural delivery and cesarean section. Independent of the type of delivery, uterine stiffness gradually increases over the first 10 days postpartum. For the myometrium, SWV shows a linear increase, while for the endometrium, SWV increased similarly (102). These results indicate progressive tissue remodeling and histological changes consistent with normal postpartum involution. Despite larger uterine diameters observed in the cesarean group, no significant differences in stiffness were found between the two groups, suggesting that tissue remodeling progresses similarly, even if macroscopic involution is delayed (102).

#### 4.4.2 Abnormal findings

##### 4.4.2.1 Pregnancy progression

One of the most common early pregnancy complications is *embryonic resorption* (104), detectable through specific ultrasonographic features. The initial signs include delayed development of the gestational sac or embryo proper (105). As resorption progresses, the yolk sac fluid becomes hypoechoic rather than anechoic, often containing echogenic particles. The embryo’s margins blur, and cardiac activity ceases, marking the transition to resorption, and the uterine wall surrounding the resorbing conceptus may bulge inward and appear hypoechoic (105). Advanced cases show lifting of the placenta and the escape of allantoic fluid, culminating in the disappearance of the embryonic structures (105). This is often associated with early embryonic death during the first trimester, and deviations in fetal biometry, such as smaller-than-expected BPD, BD, or FL for the gestational age, can be signs of abnormalities in the timing of fetal development (69, 76, 105).

*Fetal malformations*, including skeletal anomalies or organ deformities, are best observed during mid-to-late gestation when organogenesis and ossification allow clearer visualization of the fetal structures (2). Prenatal ultrasonographic evaluations allow the early identification of twin pregnancies in dogs, particularly during the initial stages of gestation when the gestational sacs are easier to count and assess individually. *Monochorionic twins*, defined by shared placental structures, can be identified through their unique features, including two umbilical cords connected to the same placenta but with separate amniotic sacs (106). This condition, which remain rare, has been associated with potential complications such as growth disparities, placental insufficiency, and risks of dystocia, and detailed evaluations can distinguish twins from singleton pregnancies with anomalies, ensuring proper prenatal care and planning for parturition (106).

*Changes in fetal fluid compartments* are another diagnostic clue. Both excess and reduced fluid volumes can indicate membrane rupture, placental dysfunction, or disturbances in fetal fluid regulation (76). *Ascites* or *hydrothorax* may appear as anechoic areas within the fetal abdomen or thorax, often associated with fetal hydrops or organ dysfunction. *Anasarca (hydrops fetalis or congenital edema)* is a rare but clinically significant condition in dogs, characterized by generalized subcutaneous edema often accompanied by pleural and/or peritoneal effusion. Affected fetuses appear markedly enlarged on ultrasonography, with hypoechoic fluid accumulation underneath the skin and within body cavities, which may also present increased extra-fetal fluid volumes. The condition can develop rapidly, even within a few days after a normal scan, and may affect one or more fetuses within the same litter (107). Due to its strong association with obstructive dystocia, prenatal diagnosis is critical for planning assisted parturition or elective cesarean section. *Hydrallantois*, a rare condition in dogs, presents as excessive fluid accumulation in the allantoic sac, leading to abdominal distension and impaired maternal health. Ultrasonographic findings include disproportionately large anechoic areas surrounding the fetuses and distended fetal membranes (108).

*Fetal distress*, often resulting from hypoxia, metabolic imbalance or placental dysfunction, is characterized by specific Doppler alterations reflecting the fetus’s attempt to adapt to compromised intrauterine conditions. Fetal heart rate (FHR) remains the pivotal indicator to assess fetal distress, even if *persistent intestinal peristalsis*, visible via ultrasonography, has been reported as an indirect marker of distress (109). Prolonged or sustained decelerations of fetal heart rate below 150 bpm in late gestation are strongly associated with severe fetal distress and carry a high risk of death within 2–3 h if not promptly addressed (76, 109). Such decelerations may result from transient events, such as uterine contractions compressing the umbilical cord or reducing placental blood flow, but when persistent, they warrant immediate clinical evaluation and/or intervention. Values between 180 and 220 bpm are more suggestive of mild stress, often reflecting a transient adaptive response rather than critical hypoxia (76). However, it is worth noting that in certain abnormal gestations, fetal heart rate does not decelerate and may instead exhibit acceleration. This pattern has been associated with poor fetal perfusion leading to low fetal weight, as the maturation of vagal cardiac control is dependent on adequate fetal growth (110). In such cases, the immature vagal tone favours a persistently higher heart rate, and this acceleration may be further sustained by the progressive rise in plasma catecholamines that occurs during hypoxia (110). Hemodynamic assessment via Doppler ultrasonography further enhances diagnostic accuracy in complicated pregnancies. Deviations from the normal pattern, such as persistently elevated RI and PI values in the uterine or umbilical arteries, suggest inadequate placental perfusion. This condition, often linked to placental issues, compromises nutrient and oxygen delivery to the fetus (89). The evaluation of ductus venosus blood flow using Doppler ultrasonography provides critical insights into fetal cardiovascular health and distress. In particular, the presence of a triphasic ductus venosus waveform, characterized by a reversed a-wave during atrial systole, has been strongly associated with neonatal mortality, reflecting compromised cardiac function, increased venous afterload, or hypoxemic conditions (111).

**Figure 10:**
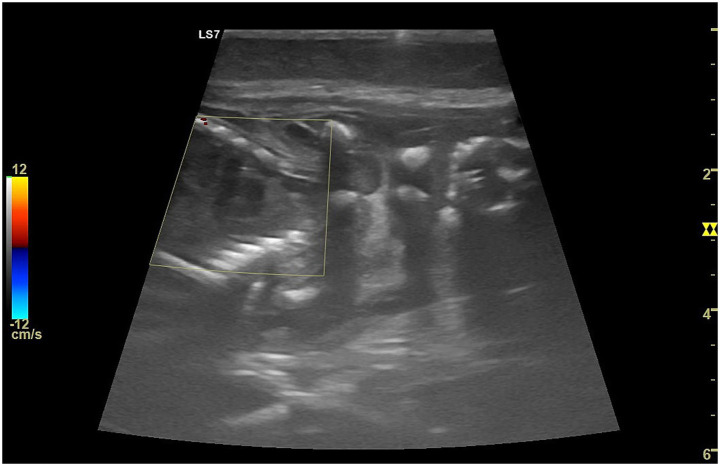
Color Doppler ultrasonographic image of a canine fetus *in utero*. No Doppler signal is detected within the fetal body or the umbilical vessels, indicating absence of detectable blood flow at the time of examination. These findings are consistent with lack of feetal circulation.

*Fetal death* is confirmed by the absence of a heartbeat when it would normally be detectable, especially if other littermates remain viable (76, 112). In cases of fetal death, Doppler ultrasonography reveals absent blood flow in the umbilical and fetal vessels, confirming the lack of circulation (Figure 10). Within 12 h after death, fetal structures become less distinct, and by 24 h, the fetus often appears as a uniformly dense, rounded structure. In cases of mummification, ossified elements such as the skull, ribs, and spine may remain visible for longer (76, 112).

##### 4.4.2.2 Post-partum complications

**Figure 11:**
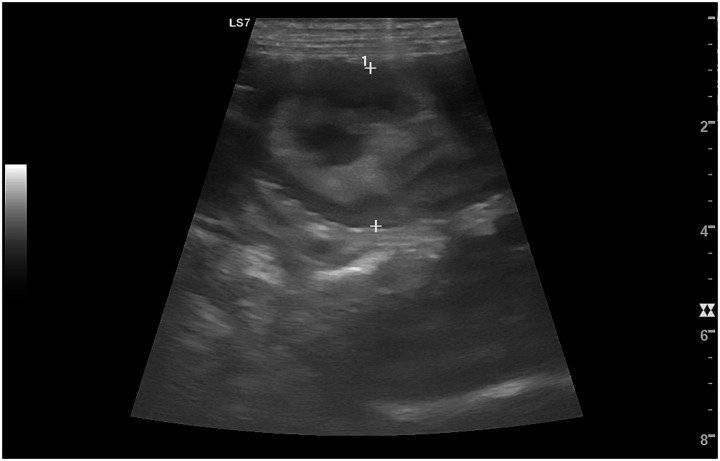
Ultrasonographic appearance of placental retention in a bitch. Longitudinal B-mode scan of the uterus, highlighted by the caliper markers (+) showing an irregular, heterogeneously hyperechoic intrauterine structure, consistent with retained placental tissue. The lesion appears poorly marginated from the surrounding endometrium and occupies part of the uterine lumen. Retained placenta is characterized by variable echogenicity and can be differentiated from normal postpartum uterine contents by its persistence, heterogeneous pattern, and association with delayed uterine involution.

After parturition, several complications may arise, often manifesting as prolonged vulvovaginal discharge. While some discharge is normal after uncomplicated delivery, its volume, color, consistency, and duration must be evaluated to distinguish physiological lochia from pathological changes (100). Common post-partum disorders include retained fetal membranes, postpartum hemorrhage, subinvolution of placental sites (SIPS), and metritis. B-mode ultrasonography is the principal tool for evaluating the postpartum uterus and identifying deviations from normal involution. *Retained fetal membranes* typically appear as persistent uterine dilation with echogenic luminal content, reflecting blood clots, tissue debris, or residual membranes (Figure 11) (36, 101).

**Figure 12:**
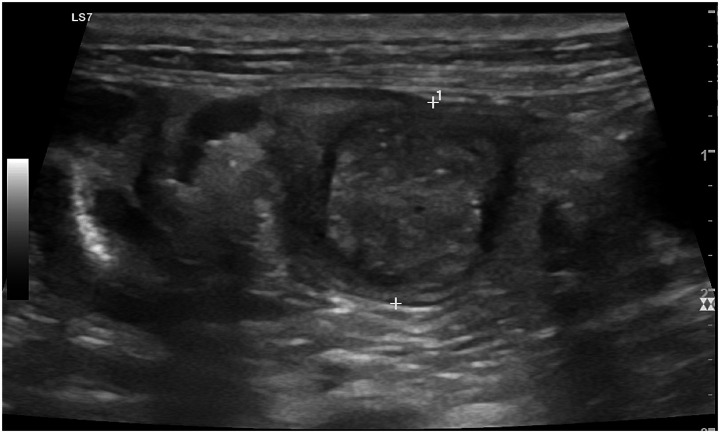
Ultrasonographic image of subinvolution of placental sites (SIPS) in a bitch. Transverse ultrasonographic view of the uterus showing marked enlargement of the uterine horn (#1), delineated by caliper markers (+). The uterine lumen appears prominently distended and filled with heterogeneous material, while the uterine wall is thickened and irregular, consistent with delayed regression of placental attachment sites, persisting beyond the normal postpartum period.

The major cause of sanguineous vaginal discharge in bitches, occurring in 10–20% of post-partum bitches from 6 weeks post-partum onwards, is *SIPS* (113). In such cases, echogenic nodules or irregular placental attachment zones may remain visible on ultrasonography, often exceeding normal dimensions beyond the expected timeline for complete involution (Figure 12) (99).

**Figure 13:**
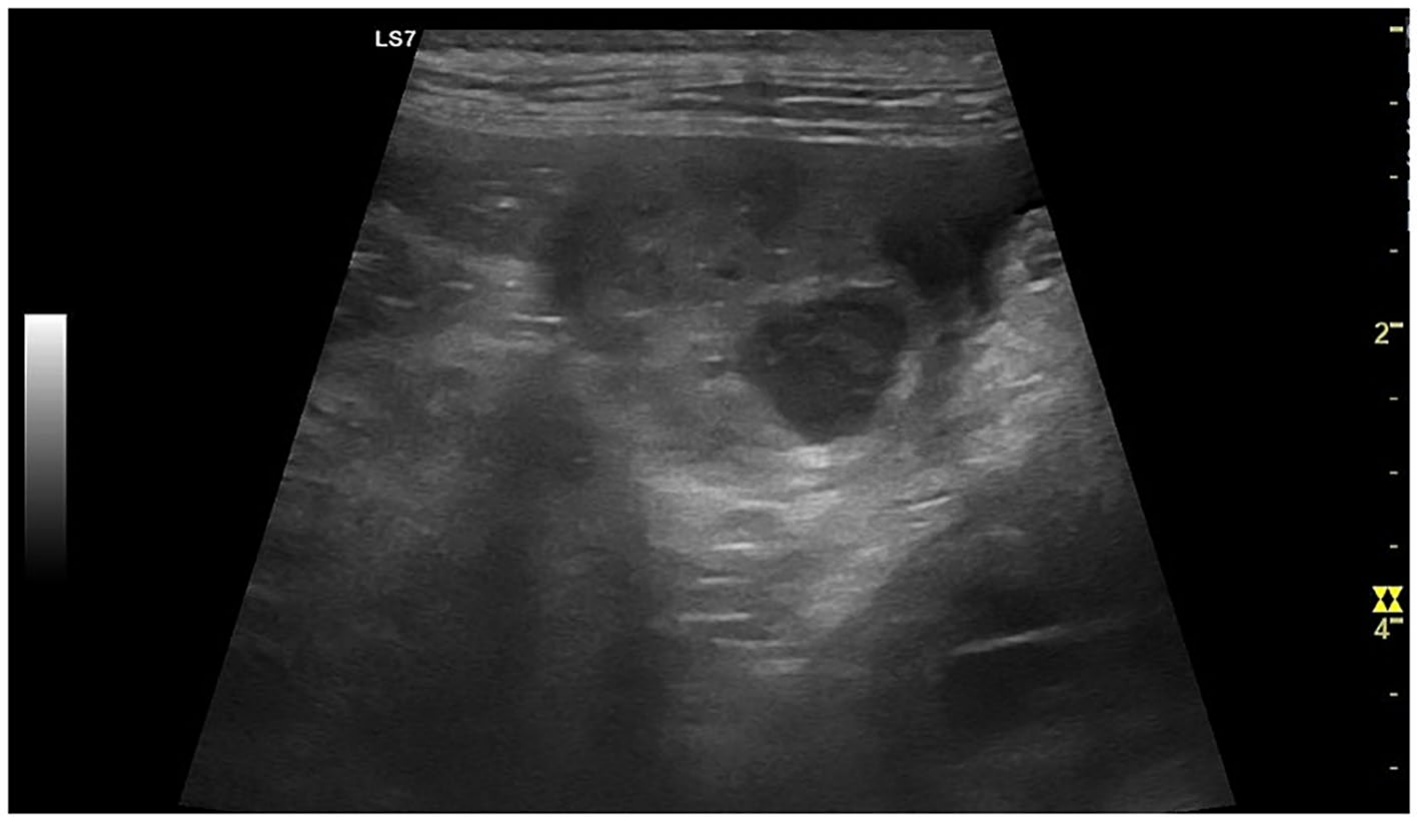
Transversal B-mode ultrasonographic image of the uterus in a bitch showing uterine horn invagination (intussusception). Within the uterine lumen, a tubular structure with a characteristic concentric layered appearance is visible. The invaginated uterine segment shows a central moderately hyperechoic core surrounded by a more hypoechoic ring, corresponding to telescoping of the mucosal and muscular layers. The outer uterine wall appears mildly thickened, consistent with local structural distortion associated with uterine intussusception.

*Metritis*, a severe postpartum complication, presents as a distended uterine lumen filled with heterogenous echogenic or hypoechoic fluid, indicating pus or necrotic material, often accompanied by thickened uterine walls (2, 100, 101). Rare conditions include *uterine invagination or intussusception*, visible as a telescoping hypoechoic structure within the lumen (Figure 9) (114) and *placenta percreta* in which trophoblasts penetrate through the myometrium, reaching the serosal layer and potentially adjacent organs, leading to severe complications such as uterine rupture and necrosuppurative metritis. On ultrasonography this appears as a fluid-filled, heterogeneous uterus with pockets of intrauterine gas, likely resulting from bacterial fermentation within the compromised uterine tissue (115), and localized wall thickening at abnormal placental attachment sites, often visualized as hyperechoic or hypoechoic zones with disrupted normal tissue architecture (Figure 13) (115). Unlike other conditions, these findings may occur without free abdominal fluid, emphasizing localized uterine pathology (115).

Doppler ultrasonography, although scarcely studied in canine postpartum pathologies such as SIPS or retained fetal membranes, provides information on uterine perfusion. Current knowledge largely derives from human medicine, where retained products of conception typically exhibit marked hypervascularity, low RI and elevated PSV, particularly in the presence of active trophoblastic tissue, which increases the risk of hemorrhage and often necessitates intervention (116, 117). By extrapolation, pathological subinvolution of placental sites in bitches would likely present with persistently increased blood flow and low RI at placental sites beyond the expected involution period. A similar hemodynamic profile can be hypothesized for postpartum metritis, which, although not yet Doppler-characterized in dogs, is expected to show hypervascularity, turbulent flow, and low-resistance indices reflecting inflammation and elevated metabolic demand (118, 119). Conversely, uterine intussusception would be anticipated to produce markedly reduced or absent perfusion due to localized vascular compromise, as suggested by analogous findings in intestinal intussusception (120).

### 4.5 Cervix

#### 4.5.1 Normal findings

**Figure 14:**
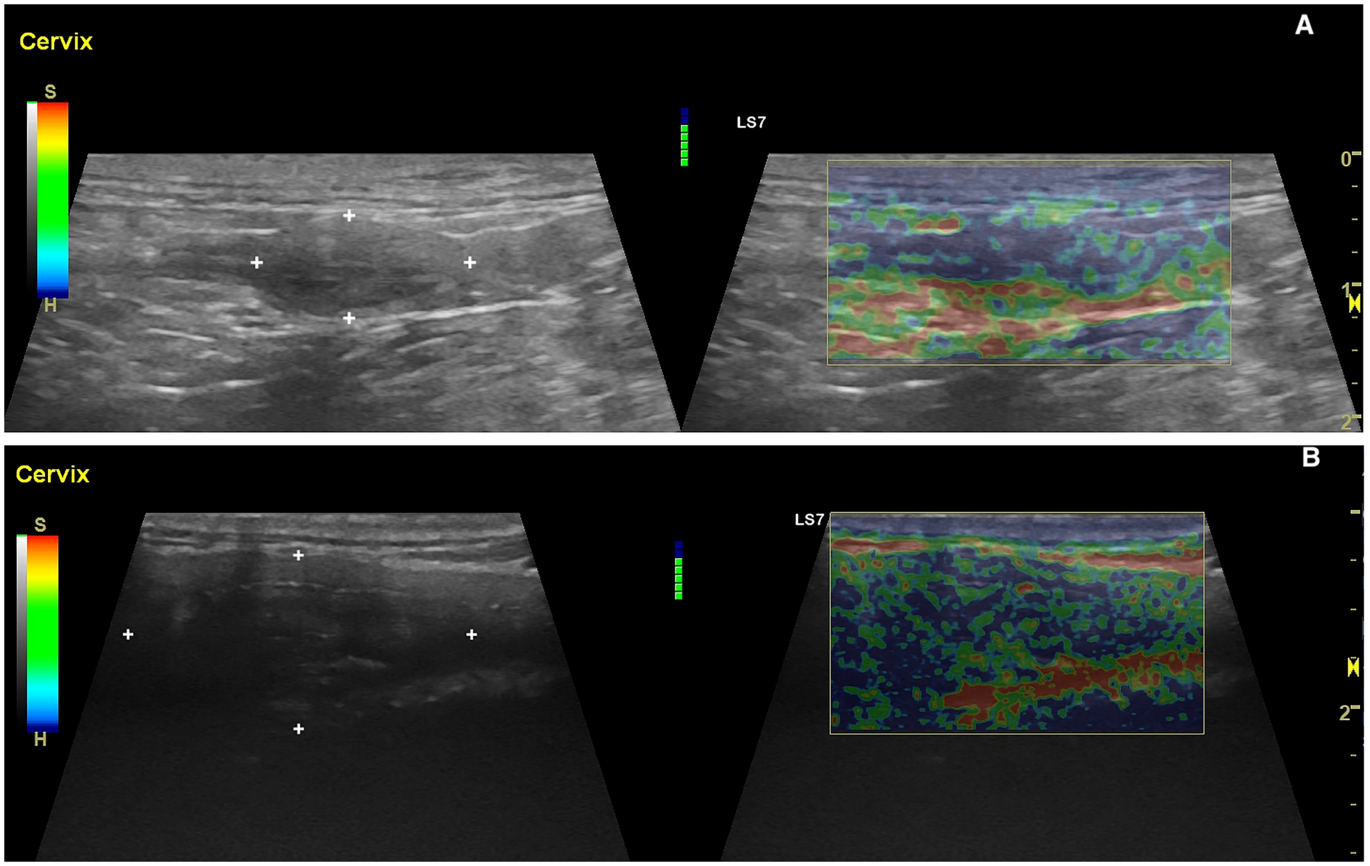
B-mode and elastographic longitudinal views of the cervix in a bitch during different stages of the estrous cycle. **(A)** Uterine cervix, outlined by caliper markers (+), during anestrus. The tissue appears hyperechoic and compact on B-mode (left), while the corresponding elastographic mode (right) shows predominant red and green areas, indicating stiffer tissue with higher elasticity index values. **(B)** Uterine cervix, identified by caliper markers, during estrus. B-mode imaging (left) reveals a more relaxed and slightly hypoechoic cervix, while elastography (right) displays a dominance of blue areas, indicating softer tissue due to organ’s relaxation.

The normal cervix, located slightly cranial to the bladder trigone, appears as a hyperechoic, linear structure in a sagittal view (2, 23). The cervical canal typically presents as a linear echogenic band, reflecting the apposed mucosal surfaces. Its dimensions and consistency vary across the estrous cycle, influenced by hormonal changes, particularly estrogen and progesterone (4). Recent studies confirmed these variations using both B-mode ultrasonography and elastography (Figures 14A,B) (4, 121). In human obstetrics and gynaecology, elastography is often combined with conventional B-mode ultrasonography to evaluate cervical length during pregnancy, enabling the identification of cervical insufficiency and associated risks of preterm birth. This combination also provides critical information on cervical dilation, thinning, and maturation during labor, facilitating timely clinical decisions regarding delivery management (122–127).

#### 4.5.2 Abnormal findings

Cervical neoplasia, though rare, remains a notable concern. Neoplastic lesions may appear as solid or mixed echotexture masses with irregular margins, often disrupting the normal cervical architecture (55). In some cases, cervical tumors may cause luminal obstruction, leading to secondary complications like hydrometra or pyometra (59). Ultrasonography can reveal associated fluid accumulation proximal to the obstruction, characterized by anechoic or hypoechoic fluid within the uterine lumen (2, 59). In human medicine, the characterization of cervical abnormalities, such as polyps, cysts, fibroids and malignant lesions, often relies on the combined evaluation of morphology and tissue stiffness (126, 128–131). Elastography further enhances diagnostic accuracy, proving effective in the early detection of cervical cancer by distinguishing invasive cervical tumours from non-invasive cervical intraepithelial neoplasia (CIN) and in predicting responses to chemoradiotherapy (126, 129).

In addition to neoplastic processes, *foreign bodies* such as grass awns can occasionally be detected ultrasonographically in the cervical region. These foreign materials typically appear as echogenic structures within the affected area, often surrounded by hypoechoic regions indicative of localized inflammation (23). Persistent foreign material can lead to chronic irritation or secondary infections, which manifest ultrasonographically as thickened cervical walls or the presence of echogenic debris (2, 23).

## 5 Future perspective

Although ultrasonographic imaging of the canine uterus has reached a high level of diagnostic refinement, several areas still offer opportunities for development. Advances in transducer technology, image resolution, and software-based quantitative analysis are expected to improve the detection of subtle structural changes and early pathological processes. Techniques such as CEUS and elastography, while promising, remain underutilized in veterinary medicine due to limited availability, lack of species-specific reference ranges, and the need for specialized training. Future research should focus on establishing normative datasets for different reproductive stages and pathological conditions, enabling more accurate interpretation and broader clinical application.

Integration of multiparametric approaches may allow simultaneous morphological, functional, and biomechanical assessment, thus enhancing diagnostic accuracy and prognostic capability. Furthermore, three-dimensional and four-dimensional ultrasonography, already established in human gynecology, could be adapted and validated for routine veterinary use, particularly in complex surgical planning and detailed fetal evaluation. Emerging trends in human reproductive imaging, such as artificial intelligence–assisted interpretation and automated volumetric analysis, also hold promise for veterinary application, potentially reducing operator dependency and improving reproducibility. Finally, collaborative multicenter studies and cross-species comparisons could accelerate the translation of novel techniques into everyday clinical practice, ultimately improving uterine health monitoring and reproductive management in the bitch.

## 6 Conclusion

The evolving landscape of ultrasonography offers promising avenues for advancing the diagnosis and management of reproductive conditions in domestic canines. Traditional grey-scale ultrasonography continues to serve as a fundamental diagnostic tool, while Doppler ultrasonography has enhanced the ability to assess vascular dynamics across various reproductive states. With its safety profile and diagnostic capabilities, CEUS and elastography hold promise for expanding its role in the assessment of reproductive pathologies in domestic animals. The introduction of 3D and 4D ultrasonography has further expanded imaging capabilities, allowing for detailed anatomical and functional assessments of the uterus and fetuses. However, the application of these advanced modalities remains underutilized in veterinary medicine, particularly for evaluating the canine uterus. Bridging this gap requires further research to establish standardized protocols, expand clinical applications, and validate the diagnostic accuracy of these technologies.
